# Braintrust: What Neuroscience Tells Us About Morality

**DOI:** 10.5964/ejop.v14i2.1589

**Published:** 2018-06-19

**Authors:** Farid Pazhoohi

**Affiliations:** a Department of Basic Psychology, School of Psychology, University of Minho, Braga, Portugal

[Other p1]Patricia Smith Churchland, professor emerita of philosophy at the University of California, San Diego, and an adjunct professor at the Salk Institute, coined “neurophilosophy,” to refer to the application of neuroscientific concepts to traditional philosophical questions. In *Braintrust: What Neuroscience Tells Us about Morality*, Churchland asks where values come from, and incorporates biological sciences with philosophy to answer the related moral questions.

In the first chapter, Churchland criticizes current conceptions of morality by asking why there are still unanswered fundamental questions in the field, including questions surrounding the nature of fairness. She believes that contemporary moral philosophy is “in peril of floating on a sea of mere, albeit confident, opinion” (p. 2) and has no relation to the current scientific findings in evolutionary biology and neuroscience. She suggests that we can answer some of the remaining moral questions by combining new findings in neuroscience, evolutionary biology, experimental psychology, and genetics within a philosophical framework.

In the second chapter, Churchland emphasizes the intricate neural circuitry of the pain and reward system corresponding to the painfulness of separation and the pleasure of company. After explaining the underlying neurobiological mechanism of attachment, she suggests that moral practices are rooted in social desires, most importantly in an attachment to family members, care for friends, and the need to belong.

The third chapter deals with the question how organisms care about others. Churchland explains how the inner state of the body is translated to the generation of motivational emotions and perceptual cues assessing the risks and opportunities of the outside world. Here pain and fear are identified as main emotions for corrective behaviors, as warning signals and as means of self-preservation. She then explains the neurobiology and the mechanisms of mate attachment, parenting behavior, and the physiology of behavioral responses. She argues that there is no need for a specific underlying mechanism to explain the neurobiology of moral behavior as “the pain of exclusion, separation, and disapproval, […] exploits, expands, and modifies what is already in place for physical pain and homeostatic emotions in premammalian species” (p. 46).

In the fourth chapter, Churchland focuses on the neurobiology of oxytocin and its effects on trust and caring relationships. She claims that morality originates in the neurobiology of attachment, and so depends on the function of the oxytocin-vasopressin network in mammals. She notes that trust between individuals has much to do with oxytocin and vasopressin. Unfortunately, while Churchland offers detailed explanations of the relationship between oxytocin in trust and cooperation (concentrating on the effect of this neurotransmitter on human and animal behaviors), she neglects the evolutionary biological point of view, thus overlooking some very important arguments.

In the fifth chapter, Churchland explains gene-behavior relationships and how changes in genes would affect behavior. She reminds the reader that the gene/behavior relationship is complicated and she provides different examples and arguments to show that the gene/behavior relationship is not straightforward. In the sixth chapter, she deals with the function of prefrontal cortex, which “yields the intelligence in human social behavior” (p. 119). Then she explores mirror neuron systems, the underlying neural mechanisms in attributing mental states to others and to oneself. Although she is an optimist in explaining the neural substrate for complex social behaviors, she acknowledges the inability of current neuroscientific findings to explain the brain’s mechanisms and functions. In addition, she is skeptical that the mirror neuron system alone could be responsible (and sufficient) for explaining human intentions.

In the seventh chapter, Churchland explains the classical notion of morality in philosophy and highlights the shortcomings of these notions in providing universal rules. She explores different ethical doctrines and concludes that “counting on pure rationality and consistency to undergird morality is mistaken” (p. 175) as all intuitions are products of the brain generated in some way by nervous systems while remaining dependent on experience and cultural practices; therefore, these intuitions cannot reveal any metaphysical truths. She rejects the idea that supernatural beings are the source of morality, as something that is good or just or right is rooted in the nature of humans, and because, likewise, it is human nature that determines whether some social practices are better than others.

Her main thesis is that morality is based on social behaviors shaped by brain processes that include caring for kin and kith, the ability to recognize and predict others’ mental states and intentions, our capacity to problem-solve in a social context, and learnt social practices. Morality, to Churchland, is “a natural phenomenon—constrained by the forces of natural selection, rooted in neurobiology, shaped by the local ecology, and modified by cultural developments” (p. 191). Therefore, the pain we avoid and the pleasure of belonging, along with the imitation of those we admire, “give rise to powerful intuitions about the absolute rightness or wrongness of classes of behavior” (p. 192).

There are some shortcomings in this book that make her thesis ambiguous and the argumentation at times unconvincing. Overall, the relationship between hormone and behavior, gene-behavior, gene-environment and the claim that the behavior is brain-based are not novel. There is a huge body of literature on these relationships, so it is not obvious from the content of the book what her thesis adds to this conversation. Churchland herself confesses this, noting that “the core of the biological approach to human morality favored in this book is not new, though my particular way of synthesizing the data and encompassing the relevant philosophical tradition may be” (p. 11). In addition, she is unsuccessful in extending the approach to the realm of philosophy, as she fails to offer a detailed explanation of morality from a philosophical point of view. At some points, Churchland discusses unrelated subjects (e.g., in chapter six she goes through a detailed description of fMRI technique function), which seem unnecessary to the overall argument, and, occasionally, paragraphs or sections lose focus. Finally, the book finishes rather suddenly, without specifically highlighting a conclusion.

Nikolaas Tinbergen, a Dutch biologist and the winner of the 1973 Nobel Prize in Physiology or Medicine, introduced four fundamental questions for an integrative explanation of behavior, that seek to, identify the function, causation, development and evolution of behavior (Tinbergen, 1963). The proximate explanation answers *how* an organism's structure functions and the ultimate question considers *why* a species evolved the adaptations it has. Churchland explains the neurobiology of caring and the underlying mechanisms of social norms and behaviors (i.e., the *how* question) but she is not successful in linking evolutionary biology with morality (i.e., the *why* question). In other words, she argues that the brain is responsible for all human emotions and hence morality, but she fails to explain why morality has been adaptive and why it evolved the way it did. However, there are successful examples of evolutionary approach to morality (Baumard, 2016; Baumard, André, & Sperber, 2013).

The potential audience of this book includes those philosophers who are not familiar with neuroscience and biology, as the biological discussions in this book are classical concepts of these fields. At the same time, it seems unlikely that a scholar with a philosophical background would find himself interested in detailed neurobiological arguments and discussions that are provided in this book. However, putting the criticisms aside, this book tries to open a new perspective to the philosophers and moral researchers and could be considered a pioneering approach in separating morality from the body of philosophy. Educated general readers interested in the neurobiology and the underlying mechanisms of cooperation, attachment, and pair bonding might also find the book interesting.
